# Simultaneous Quantification of 14 Compounds in *Achillea millefolium* by GC-MS Analysis and Near-Infrared Spectroscopy Combined with Multivariate Techniques

**DOI:** 10.1155/2021/5566612

**Published:** 2021-05-24

**Authors:** Lan-Ping Guo, Jian Yang, Li Zhou, Sheng Wang, Chuan-Zhi Kang, Christian W. Huck

**Affiliations:** ^1^National Resource Center for Chinese Materia Medica, China Academy of Chinese Medical Sciences, State Key Laboratory Breeding Base of Dao-di Herbs, Beijng 100700, China; ^2^Head of Spectroscopy Group, Institute of Analytical Chemistry and Radiochemistry, Leopold-Franzens University, Innrain 80, Innsbruck 6020, Austria

## Abstract

The proposed work is focused on the simultaneous quantification of 14 compounds in the medicinal plant *Achillea millefolium* based on Near-Infrared Spectroscopy (NIR). The regression model of single-compound models (SCMs) and multicompound model (MCM) were created by partial least-squares regression (PLSR). Also, these models were calibrated by gas chromatographic mass spectroscopy (GC-MS). The results showed that the averaged standard errors of prediction (SEP) for the SCMs and MCM were 0.49 and 0.62, respectively, and most of the 14 compounds were significantly correlated. 43 correlations were significant at the 0.01 level (47.25% of the total), and 11 correlations were significant at the 0.05 level (12.09% of the total). The first three principal components (PCs) of principal component analysis (PCA) can explain >78% of the total variance. According to the component matrix and the communality table, octadecanoic acid has the largest influence on PC 1 (extraction squared = 46.72%), whose extraction was 0.932. The communality of neophytadiene, Z,Z,Z-9,12,15-octadecatrienoic acid, and oleic acid was also found to be large, whose extractions were 0.955, 0.937, and 0.859, respectively. These results indicate that if one compound shows a linear relationship with the NIR absorbance signal (SCM) also, an MCM can be created due to the close interrelations of these compounds. In this context, the present work highlights a suitable sample preparation technique to perform NIR analysis of raw plant material to benefit from robust and precise calibrations. To sum up, this NIR spectroscopic approach offers a precise, rapid, and cost-effective high-throughput analytical technique to simultaneously and noninvasively perform quantitative analysis of raw plant materials.

## 1. Introduction

Currently, there is a growing need for the analysis of medicinal plants because they contain a large number of beneficial medicinal compounds. *A. millefolium*, a widely distributed medicinal plant in Europe and Asia, is extensively used as a folk medicine because of its multiple pharmacological activities, and its essential oils are important for the anti-inflammatory activities of plants [1–3]. To quantitatively determine one compound in a medicinal plant, often the information about the remaining compounds is lost due to the extraction of only one single compound, which, however, may be the key for the therapy effect [4, 5]. Over the years, these research studies were focused on fingerprint technology, such as high-performance liquid chromatography (HPLC), gas chromatography (GC), high-performance thin-layer chromatography (HPTLC), capillary electrophoresis (CE), and nuclear magnetic resonance (NMR), which are helpful to give an overall understanding of the chemical active ingredients [6–10]. In doing so, it has to be considered that fingerprint techniques are commonly complex and very specific for only one or few compounds which makes single-spectrum-based fingerprint techniques not only time consuming but also hard to standardize [11]. To simultaneously determine multiple compounds, other analytical techniques, oftentimes combined with multivariate techniques that generate a series of fingerprint spectra, have to be applied. The near-infrared (NIR) region expands from 4000 to 12800 cm^−1^ (2500–780 nm), which covers the overtone and combination transitions of the C-H, O-H and N-H groups. Compared with midinfrared spectra, NIR absorption bands are weaker and more difficult to identify due to the higher level of excitation bonds [12]. The molecular overtone and combination bands in the NIR are typically broad and overlapping leading to complex spectra mixes. Since it is very difficult to assign characteristic features to specific chemical components, multivariate analysis (MVA) techniques, e.g., regression (PLSR), principal component regression (PCR), or multiple linear regression (MLR), are often employed to extract the desired chemical information and to bring out hidden data structures. These days, an increasing number of research is focused on the chemical assembly of the medicinal plants to quantify the relevant compounds in the samples by NIRS [13, 14], for example, *Cortex phellodendri* [15], *Magnolia officianlis* [16], *Piper methysticum* Forst. f. [17, 18], and *American ginseng* [19]. The noninvasive character of NIR spectroscopy for determining medicinal relevant compounds shows many advantages to other techniques, such as hardly any need for sample preparation and the possibility to perform outdoor analysis with commercially available hand-held instruments [20,21]. Since NIRS is often combined with MVA and statistics, parameters such as the right choice of the calibration set (training set) and the validation set (test set) samples, data pretreatments, and statistic methods for constructing the model play an important role for creating a suitable quantitative model [22–25]. Only few studies focus on the interrelation of the chemical active compounds which might be one of the most important factors when talking about herbal medicine. Most of the time, only one regression model for each property, which can be called a single-compound model (SCM), is used. This means, to quantify many compounds in a medicinal plant, one has to build as many regression models as there are compounds present in the sample. To simultaneously determine more than one compound, a multiple compound model (MCM) has to be generated. Furthermore, a MCM can reflect the interrelationship of all the compounds in the samples by only one measurement. To sum up, the main objective of this study was to quantify each of the 14 main compounds in *A. millefolium* by NIRS and to compare the single-compound data with the multicompound data evaluation technique. Diverse sample preparation procedures are reviewed, and the different multivariate data evaluation approaches are discussed in detail.

## 2. Materials and Methods

### 2.1. Sample Preparation of *Achillea millefolium*

*A. millefolium* plants were collected around Innsbruck (Austria, Europe). Each sample consisted of 5 individual plants, 36 samples in total. Twelve samples were dried in an oven at 40°C, and the remaining 24 were dried at room temperature. After the drying process, flower heads were cut off and grinded by using a roll cut machine (IKA/ULTRA TURRAX/Tube drive, Staufen, Germany) to about 1 mm. All the grinded samples were stored in an exsiccator prior to NIR analysis.

### 2.2. NIR Spectroscopy

NIR Fourier-Transform spectrometers (FT-NIR; Büchi, Flawil, Switzerland) were used to measure the NIR spectra (4000 to 10000 cm^−1^) of samples. Spectra were recorded in the diffuse reflection mode by using an integrating sphere device (Büchi). Each of the 36 samples was measured three times, leading to 108 NIR spectra, and analyzed by Chemometric software NirCal 4.21 (Büchi). These spectra were randomly divided into two parts, a learning set (67%, c-set) and test set (33%, v-set). The reflection spectra were transposed to the log (1/R) absorbance spectra followed by various data pretreatments to correct for offset effects due to an inhomogeneous particle size distribution. Partial least-squares regression (PLSR) and principal component regression (PCR) analysis were implemented to build the models.

### 2.3. GC-MS Analysis

The dried flower heads were extracted 3 times with CH_2_Cl_2_ (1 : 10 w/v) and ultrasonicated for 10 min. After evaporation of the solvent, the supernatant was transferred to a volumetric flask; n-Heptanol was used as an internal standard. The extracted compounds were identified by GC-MS using an Agilent 6890 Network GC system MSD ChemoStation (Palo Alto, US). Column: MS quartz capillary column (0.25 mm I.D × 30 m × 0.25 *μ*m). Carrier gas: helium, 2 mL/min; split ratio: 1 : 10; temperature program: 60°C to 180°C at 5°C/min and 180°C to 280°C at 2.5°C/min. Electron impact (EI) spectra were obtained at −70 eV. The search libraries were NIST02, Wiley7n, and Flavor2.

### 2.4. Quantitative Data Analysis

#### 2.4.1. NIRS Model Evaluation

The optimum number of factors for building the models was obtained by the predicted residual error sum of squares (PRESSs) function given as

(1) $$\begin{array}{c} \text{PRESS}=\sum {\left(x_{n}-y_{n}\right)}^{2}, \end{array}$$

where *x*_*n*_ is for predicted values and *y*_*n*_ for reference values.

The optimum regression models were evaluated by the following calculated values:

(i) Bias of the c-set and the v-set, which show the deviation between the values of predicted and actual; it is naturally zero after bias correction.
  (2) $$\begin{array}{c} \text{BIAS}=\frac{1}{N}\sum \left(x_{n}-y_{n}\right). \end{array}$$
(ii) The c-set SEE and the v-set SEE (SEP), which show the precision of the regression models for the c-set and the v-set, respectively.
  (3) $$\begin{array}{c} \text{BIAS}=\frac{1}{N}\sum \left(x_{n}-y_{n}\right). \end{array}$$
(iii) Consistency, which shows the robustness of the regression models; it should approach 100. Consistency = SEE/SEP × 100.
(iv) Regression coefficient (*R*^2^) of the c-set and the v-set, which will show the relation of the predicted values to the actual values; *R* should approach 1.
(v) Regression intercepts and slopes of the c-set and the v-set.

#### 2.4.2. Comparison of the PLS Models

A paired t-test was conducted to compare the difference between the SCM regression models and the MCM. ANOVA was used to compare the differences of the varying sample preparations (air-and-oven-dried, oven-dried and air-dried). The homogeneity of the variances was tested by Levene statistic, and multiple comparisons were conducted by LSD when the equal variance was assumed to be equal and by Tamhane in case of nonequal variance. Pearson bivariate correlation, principal component analysis (PCA),, and hierarchical cluster analysis were conducted to find the inner relationship among the 14 compounds.

## 3. Results and Discussion

Comparison of the single-compound models (SCMs) and the multiple compound model (MCM).

44 compounds were identified by GC-MS (data not showed here), whereas those 14 which seem to be of particular interest in herbal medicine were used for creating the PLS models (Table 1).

**Figure 1:**
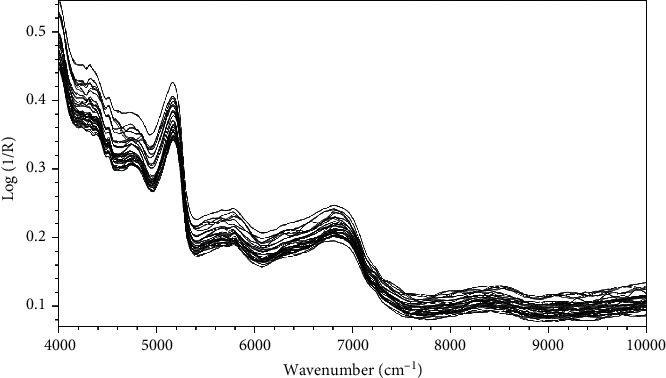
NIR absorbance spectra of the grinded *A. millefolium* plants.

**Figure 2:**
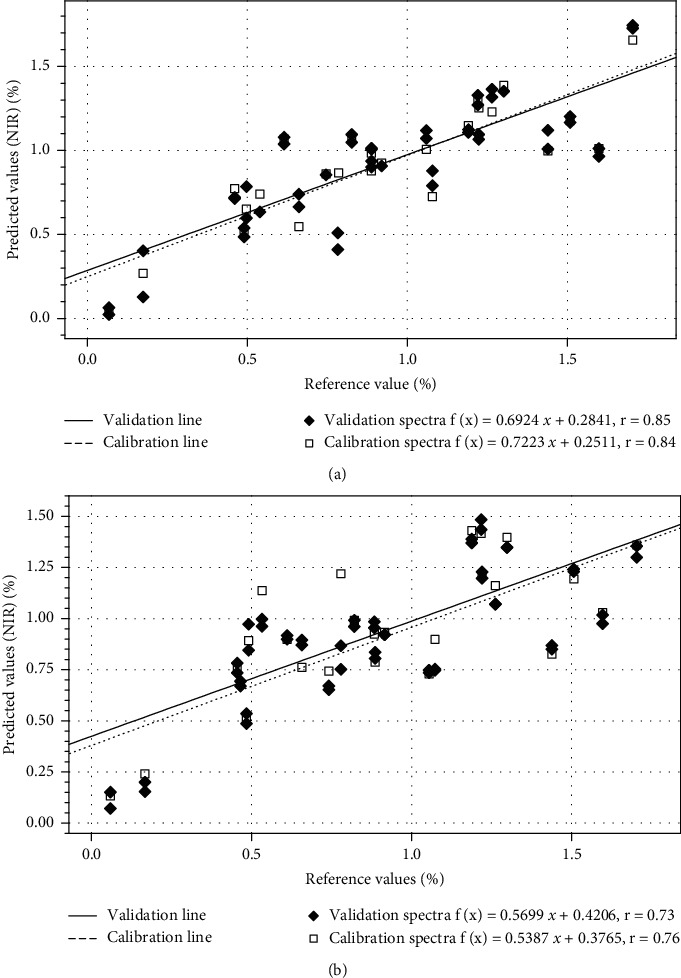
PLS regression lines of the SCM (a) and the MCM (b) model for determining the n-decanoic acid content in *A. millefolium*.

The 108 log (1/*R*) NIR spectra (Figure 1) were used for creating the 17 PLSR models (Table 2, Figures 2(a) and 2(b)), including the 14 models for each single compound 1 to 14 and the three models for air-dried samples, oven-dried samples, and oven-and-air-dried samples, respectively.

As can be gathered from Table 2, varying wavelengths, data pretreatments, calibration methods, and factors were used to build the best and most specific regression model for each single compound. Wavelengths for different compounds did not vary much; most of them focus on a broad wavenumber range from 4596 to 9000 cm^−1^. MSC was used as a data pretreatment for 9 of the 17 models to suppress unwanted scatter effects due to different particle sizes present in samples. Since PLS is well suited for coupling digital filtering [24], it was indicated that the NIRS data show a collinear relationship to some extent. Coupling digital led to a low consistency in all cases, which is needed for MSC as a data pretreatment. MSC turned out to reducing the calibration factors, or simplifying the regression model, and increasing the consistency (robustness) of the models. [26]. The average number of factors after the MSC data pretreatment was 6, which is the lowest amount of average factors for all data pretreatments applied. The PLS model evaluation showed an average SEE of 0.35 for the SCM and 0.56 for the MCM whereas the average SEP showed 0.49 and 0.62, respectively. The average consistencies of the line from regression for the SCM and the MCM are 69.01 and 92.34. The average R2 for the c-set for the SCM and the MCM is 0.93 and 0.83, while the average R2 of the v-set for the SCM and the MCM is 0.89 and 0.82, respectively (Table 3). It was shown that both the SCM and the MCM have high R2 and low SEE and SEP, which is an indication for the high prediction abilities of the individual SCMs and the simultaneous determination by means of the MCM. The paired t-test showed significant differences between each parameter for the SCM and the MCM, except the bias, although both the SCM and the MCM are well suited for the prediction of unknown samples. The SEE and SEP of the 14 compounds calibrated by the SCM were lower than those obtained by the MCM, and the R2 of both the c-set and v-set of the 14 compounds calculated by the SCM was higher than that by the MCM. In detail, from the SCM to the MCM, the SEE increased at 0.22 (*p* < =0.01) and the SEP at 0.12 (*p* < =0.01). The consistency increased at 23.33 (*p* < =0.05) while the R2 decreased at 0.10 and at 0.07 for the c-set and the v-set, respectively. These results denote that the MCM, representing 14 compounds, shows, on one hand, a higher robustness of the regression model but reduces the precision and prediction ability on the other hand. In other words, one has to decide whether to perform simultaneous, fast, and robust but not very precise analysis (MCM) or to perform very precise single-compound analysis by implementing a single-compound model.

### 3.1. Influence of the Sample Sets on the Regression Models

PLS models of the 14 compounds for different sample pretreatments were built and compared to the MCMs (Table 2). The S_air-oven_ models showed a higher SEE (average = 0.85), SEP (average = 0.87), consistency (average = 98.04), and intercept for the c-set and the v-set (average = 1.13 and 1.16), but a lower v-set bias (average = 0.02), *R*^2^ (v-set = 0.72, c-set = 0.73), and slopes (v-set = 0.54, c-set = 0.53) than both S_oven_ and S_air_ (Tables 3 and 4). For the S_oven_ and the S_air_ regression line, there were no significant differences between most of the parameters except the consistency, which is higher in S_air_ (92.34) than in S_oven_ (61.98) (*p* < =0.01) and the v-set *R*^2^ of S_air_ (0.83) and S_oven_ (0.92) (*p* < =0.05). The conducted ANOVA showed most parameters of the v-set (except v-set bias) were not significantly different with the other parameters. That means that many samples with great variations can increase the robustness of the regression models, but reducing the *R*^2^ and the precision as a consequence. All the regression models were almost equal in the prediction ability since there were no differences in the v-set evaluation. One thing that has to be considered is that all the 16 regression models (14 SCMs, S_air_ and S_oven_) were employed by PLS, and only the S_air-oven_ showed significantly better regression parameters by employing PCR, which maybe relevant to the greater variation but lower collinearity in the calibration set of S_air-oven_.

### 3.2. Interrelation of the 14 Main Compounds in *Achillea millefolium*

Our research brought up the following question: why can 14 compounds with different properties be quantified by only one NIR regression model?

It is implied that there have to be some internal relationships between the 14 compounds. In order to expose these, Pearson bivariate correlation, PCA, and hierarchical cluster analysis were conducted on the 14 compounds. Pearson bivariate correlation analysis showed that most of the 14 compounds were significantly correlated. 43 correlations were significant at the 0.01 level (47.25% of the total), 11 correlations were significant at the 0.05 level (12.09% of the total), and 37 correlations were not significant (40.66% of the total) as can be seen in Table 5.

**Figure 3:**
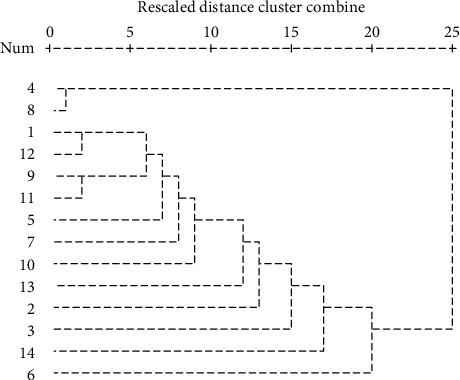
Dendrogram of the 14 compounds using average linkage (within group), z scores standardized.

The PCA showed that 3 PCs explain >78% of the total variance of the 14 compounds. According to the component matrix and communality tables (not shown here), octadecanoic acid (C12) is mainly related to principal component (PC) 1 (extraction squared is 46.72%), whose communality extraction is 0.932. The communalities of C4 (neophytadiene), C8 ((Z,Z,Z)-9,12,15-octadecatrienoic acid), and C9 (oleic acid) were also high, whose extractions were 0.955, 0.937, and 0.859, respectively (Table 6). These results corresponded to the cluster analysis in Figure 3, which showed that C1, C2, C3, C5, C6, C7, and C1 were close to C9 and C12, but far from C4 and C8. In other words, C4, C8, C9, and C12 represented the main variances of the 14 compounds.

### 3.3. Choosing the Right Sample Sets

It is a fact that different sample sets lead to different NIR regression models. Each of them may have different characteristics even if they all work well [27]. Large sample varieties and numbers can help building high robust models. In contrast, a suitable sample pretreatment procedure leads to homogenous sample sets that result in higher-precision calibration models. Careful preparation of the validation set before analysis leads to much more precise predictions and minimizes the need for spectral data pretreatment. The more homogenous the c-set and the v-set samples, the better the model and the predictions will be. Careful development of the c-set and v-set samples is crucial for near-infrared spectroscopic analysis for quantifying medicinal plants with varying compounds.

## 4. Conclusions

Both one regression model for one compound (SCM) or one regression model for multiple compounds (MCM) can be used to quantify the chemical compounds in *A. millefolium*. The former approach showed a higher *R*^2^ for the c-set and the v-set and lower SEE and SEP than the later approach that, in contrast, showed a higher consistency than the former approach. It seemed that although the *R*^2^ decreased and the SEE and the SEP increased, the MCM with many compounds brings out some internal relations of the compounds present in the samples. The MCM showed an increased robustness whereas the precision decreased. In our opinion, a combined use of a SCMs and a MCM is well suited to quantitatively analyse *A. millefolium* as well as other medicinal plants. This method has some similarity to chemical fingerprint methods, but presents itself simpler in operation than the other methods [28]. Theoretically, the MVA-supported NIR technique could merge data arising from different chemical methods, such as GC-MS, HPLC/HPLC−MS, HPCE, and TLC, to create a big complex model, which would look like a multidimensional fingerprint. Generally, the construction of an MCM needs large sample amount, the more, the more robust, but as soon as it is established, it can help save much time and make working more cost effective.

## Figures and Tables

**Table 1:** 14 selected compounds in *A. millefolium* which were used for building the NIR regression models.

Compound	*Rt* (min)	Name	Molecular formula	Molecular weight	Match quality	Relative content (mean %)	SE	SD
C1	20.4	n-Decanoic acid	C_10_H_20_O_2_	172.15	98	1.17	0.1	0.6
C2	23.9	2,5-bis (1,1-Dimethylethyl)-phenol	C_14_H_22_O	206.17	91	1.17	0.2	1.1
C3	31.1	cis,cis-7,10,-Hexadecadienal	C_16_H_28_O	236.21	99	0.80	0.1	0.6
C4	33.2	Neophytadiene	C_20_H_38_	278.3	99	2.44	0.4	2.6
C5	36.5	n-Hexadecanoic acid	C_16_H_32_O_2_	256.24	98	4.97	0.3	2.1
C6	41.2	3a,5,5a,9,9a,9b-Hexahydro-9-hydroxy-5a,9-dimethyl-3-methylene-naphtho[1,2-b]furan-2,6(3H,4H)-dione	C_15_H_18_O_4_	262.12	94	1.42	0.1	0.9
C7	41.7	(Z,Z)-9,12-octadecadienoic acid	C_18_H32O2	280.24	99	2.17	0.2	1.1
C8	41.8	(Z,Z,Z)-9,12,15-octadecatrienoic acid	C_18_H_30_O_2_	278.23	90	2.47	0.4	2.2
C9	42.0	Oleic acid	C_18_H_34_O_2_	282.26	97	3.07	0.2	1.3
C10	42.1	2-Methyl-Z,Z-3,13-octadecadienol	C_19_H_36_O	280.28	93	2.40	0.2	1.1
C11	42.3	Z,E-2,13-octadecadien-1-ol	C_18_H_34_O	266.26	99	1.34	0.1	0.6
C12	42.8	Octadecanoic acid	C_18_H_36_O_2_	284.27	99	2.17	0.2	1.0
C13	56.0	gamma-Sitosterol	C_29_H_50_O	414.39	99	3.72	0.4	2.6
C14	70.5	Stigmastan-3,5-dien	C_29_H_48_	396.38	98	2.50	0.2	1.4

**Table 2:** Comparison of the optimized parameters for developing the NIR single-compound regression models (SCM) and the multiple compound regression model (MCM).

Sample preparation^1^	Model	Compound	*n* ^2^	Wavenumber (cm^−1^)	Data pretreatment	Method	Factors
S_air_	SCM	C1	52/26	4008–9996	MSC	PLS	5
		C2	52/26	4008–9996	MSC	PLS	6
		C3	52/26	4008–9996	MSC	PLS	7
		C4	52/26	4596–9996	MSC	PLS	6
		C5	52/26	4008–9996	MSC	PLS	7
		C6	52/26	4008–9996	MSC	PLS	11
		C7	52/26	4008–9996	Smoothing (Savitzky–Golay, 9 points)	PLS	8
		C8	52/26	4596–9996	MSC	PLS	6
		C9	52/26	4440–9000	First derivative (Savitzky–Golay, 9 points); normalization to unit length	PLS	11
		C10	52/26	4440–9000	First derivative (Savitzky–Golay, 9 points); normalization to unit length	PLS	5
		C11	52/26	4440–9000	First derivative (BCAP); normalization by closure	PLS	11
		C12	52/26	4008–9996	MSC	PLS	6
		C13	52/26	4008–9996	MSC	PLS	6
		C14	52/26	4596–9996	Log (1/*R*); normalization by closure; second derivative (Savitzky–Golay, 9 points)	PLS	12

S_air_	MCM	C1–14	52/26	4008–9996	MSC	PLS	8
S_oven_		C1–14	24/12	4596–9996	First derivative (BCAP); normalization by closure	PLS	5
S_air-and-oven_		C1–14	76/38	4008–9996	Normalization by closure	PCR	12

^1^ *S*_air_ = air-dried *A. millefolium*; *S*_oven_ = oven-dried *A. millefolium*; S_air-and-oven_ = mixture of air-dried and oven-dried *A. millefolium*, ^2^sample number in the calibration set/sample numbers in the validation set.

**Table 3:** Comparative statistics between 14 compounds represented by 14 regression models (SCM) and 14 compounds represented one regression model (MCM).

Compounds	*n* ^1^	Bias				SEE		SEP		Consistency^3^		*R* ^2^				Intercept				Slope			
		C_set_^2^		V_set_		C_set_		V_set_				C_set_		V_set_		C_set_		V_set_		C_set_		V_set_	
		SCM	MCM	SCM	MCM	SCM	MCM	SCM	MCM	SCM	MCM	SCM	MCM	SCM	MCM	SCM	MCM	SCM	MCM	SCM	MCM	SCM	MCM
C1	52/26	0	0	−0.01	−0.03	0.23	0.28	0.23	0.29	98.9	94.5	0.85	0.76	0.85	0.74	0.25	0.38	0.28	0.42	0.72	0.58	0.69	0.57
C2	52/26	0	0	−0.02	−0.02	0.14	0.18	0.16	0.19	90.23	93.33	0.93	0.88	0.91	0.86	0.07	0.12	0.11	0.17	0.86	0.77	0.81	0.71
C3	52/26	0	0	0.01	−0.01	0.11	0.2	0.16	0.22	72.03	90.95	0.98	0.92	0.96	0.91	0.03	0.1	−0.01	0.11	0.95	0.85	0.99	0.86
C4	52/26	0	0	0	0.02	0.52	0.61	0.65	0.82	80.07	74.76	0.98	0.98	0.98	0.96	0.09	0.13	0.12	0.14	0.97	0.96	0.96	0.94
C5	52/26	0	0	−0.23	−0.25	1.07	1.29	1.16	1.37	92.91	94.07	0.85	0.78	0.83	0.75	1.21	1.74	1.69	2.23	0.72	0.6	0.67	0.55
C6	52/26	0	0	0.05	0.04	0.09	0.46	0.21	0.48	40	95.91	0.99	0.81	0.96	0.8	0.01	0.41	0.02	0.39	0.99	0.66	0.94	0.64
C7	52/26	0	0	−0.11	−0.1	0.48	0.7	0.62	0.73	77.53	96.37	0.9	0.78	0.84	0.76	0.38	0.82	0.68	0.97	0.82	0.6	0.72	0.58
C8	52/26	0	0	−0.12	−0.07	0.47	0.56	0.58	0.75	80.88	75.39	0.98	0.97	0.97	0.95	0.09	0.13	0.24	0.22	0.96	0.95	0.95	0.94
C9	52/26	0	0	−0.15	−0.17	0.15	0.82	0.66	0.83	23.09	99.02	0.99	0.73	0.85	0.73	0.05	1.31	0.59	1.58	0.98	0.53	0.84	0.5
C10	52/26	0	0	−0.12	−0.12	0.33	0.45	0.37	0.47	88.85	94.88	0.92	0.84	0.9	0.83	0.33	0.62	0.64	0.86	0.85	0.71	0.76	0.66
C11	52/26	0	0	−0.08	−0.1	0.09	0.36	0.32	0.37	28.57	98.91	0.99	0.77	0.84	0.77	0.03	0.5	0.29	0.66	0.97	0.59	0.83	0.54
C12	52/26	0	0	−0.07	−0.1	0.44	0.54	0.47	0.57	93.21	95.83	0.85	0.77	0.83	0.75	0.51	0.77	0.62	0.94	0.73	0.59	0.7	0.55
C13	52/26	0	0	−0.17	−0.13	0.69	0.85	0.82	0.9	84.45	94.32	0.86	0.79	0.81	0.77	0.73	1.1	1.31	1.59	0.75	0.62	0.61	0.5
C14	52/26	0	0	−0.15	−0.09	0.07	0.59	0.48	0.63	15.37	94.47	1	0.85	0.91	0.84	0.01	0.57	0.37	0.62	1	0.73	0.89	0.74

Mean		0	0	−0.08	−0.08	0.35	0.56	0.49	0.62	69.01	92.34	0.93	0.83	0.89	0.82	0.27	0.62	0.5	0.78	0.88	0.7	0.81	0.66
SD		0	0	0.08	0.08	0.29	0.29	0.28	0.32	29.01	7.6	0.06	0.08	0.06	0.08	0.35	0.49	0.49	0.64	0.11	0.14	0.12	0.15
SE		0	0	0.02	0.02	0.08	0.08	0.08	0.08	7.75	2.03	0.02	0.02	0.02	0.02	0.09	0.13	0.13	0.17	0.03	0.04	0.03	0.04

Mean paired differences				0		−0.22		−0.12		−23.33		0.1		0.07		−0.35		−0.28		0.18		0.15	
T				−0.37		−4.29		−6.72		−2.72		5.1		7.12		−4.07		−4.14		5.1		5.7	
Sig. (2-tailed)				0.72		0^5^		0^5^		0.02^4^		0^5^		0^5^		0^5^		0^5^		0^5^		0^5^	

^1^Number of samples in the calibration set/number of samples in the validation set; ^2^a test of variances cannot be performed for c-set bias because the sum of weights is zero, ^3^consistency = (SEE/SEP)*∗*100; ^4^the mean difference is significant at the 0.05 level; ^5^the mean difference is significant at the 0.01 level.

**Table 4:** Comparison of the regression parameters for the SCMs obtained by different sample treatments.

Sample^1^	Compounds	*n* ^2^	Bias		SEE	SEP	Consistency^4^	*R* ^2^		Intercept		Slope	
			c-set^3^	v-set	c-set	v-set		c-set	v-set	c-set	v-set	c-set	v-set
**S**_**oven**_	C1	24/12	0	−0.06	0.09	0.21	43.94	0.98	0.92	0.06	0.63	0.97	0.69
	C2	24/12	0	−0.11	0.4	0.46	87.43	0.79	0.72	0.99	1.76	0.62	0.39
	C3	24/12	0	−0.01	0.15	0.22	68.76	0.96	0.94	0.07	0.28	0.93	0.75
	C4	24/12	0	−0.05	0.23	0.36	64.41	0.92	0.84	0.22	0.6	0.85	0.62
	C5	24/12	0	−0.33	0.4	0.87	45.93	0.96	0.81	0.44	3.47	0.93	0.51
	C6	24/12	0	−0.16	0.47	0.69	69.14	0.8	0.55	0.66	1.66	0.64	0.23
	C7	24/12	0	−0.18	0.33	0.51	63.81	0.94	0.88	0.3	1.07	0.87	0.63
	C8	24/12	0	−0.11	0.44	0.62	71.83	0.92	0.88	0.37	1.02	0.85	0.65
	C9	24/12	0	−0.02	0.31	0.6	51.48	0.97	0.88	0.24	1.31	0.93	0.65
	C10	24/12	0	−0.35	0.67	0.84	80.15	0.85	0.86	0.79	2.09	0.73	0.43
	C11	24/12	0	−0.1	0.23	0.37	62.43	0.92	0.85	0.25	0.94	0.84	0.47
	C12	24/12	0	−0.17	0.21	0.37	56.16	0.97	0.93	0.17	1.22	0.94	0.64
	C13	24/12	0	−0.08	0.61	2.67	22.73	0.99	0.72	0.16	3.78	0.97	0.36
	C14	24/12	0	0.08	0.65	0.82	79.51	0.86	0.77	0.86	1.38	0.74	0.59

**S**_**air-oven**_	C1	76/38	0	0.03	0.35	0.35	98.94	0.81	0.81	0.41	0.39	0.65	0.65
	C2	76/38	0	0.01	0.34	0.34	97.44	0.95	0.95	0.11	0.1	0.9	0.9
	C3	76/38	0	0.02	0.36	0.37	96.14	0.77	0.76	0.32	0.3	0.59	0.59
	C4	76/38	0	−0.14	1.05	1.15	91.54	0.91	0.9	0.42	0.59	0.83	0.82
	C5	76/38	0	0.05	1.69	1.71	98.93	0.57	0.59	3.35	3.32	0.32	0.32
	C6	76/38	0	0.02	0.5	0.47	105.94	0.81	0.84	0.5	0.5	0.65	0.64
	C7	76/38	0	0.01	0.91	0.95	96.12	0.5	0.48	1.62	1.66	0.25	0.23
	C8	76/38	0	−0.16	0.98	1.08	90.85	0.89	0.87	0.52	0.69	0.79	0.78
	C9	76/38	0	0.01	1.08	1.11	97.46	0.49	0.49	2.33	2.37	0.24	0.23
	C10	76/38	0	0	0.76	0.76	100.48	0.69	0.72	1.24	1.32	0.48	0.46
	C11	76/38	0	−0.01	0.48	0.5	97.06	0.57	0.57	0.91	0.96	0.32	0.29
	C12	76/38	0	0.04	0.74	0.73	100.28	0.63	0.66	1.3	1.28	0.4	0.39
	C13	76/38	0	0.11	1.84	1.83	100.34	0.7	0.72	1.91	1.91	0.48	0.46
	C14	76/38	0	−0.02	0.79	0.79	101.05	0.8	0.82	0.89	0.84	0.64	0.67

**S**_**air**_	**Mean**	—	**0**	**−0.08a**	**0.56ab**	**0.62**	**92.34A**	**0.83Ab**	**0.82**	**0.62ab**	**0.78**	**0.70Aa**	**0.66**
**S**_**oven**_	**Mean**	—	**0**	**−0.12Aa**	**0.37b**	**0.69**	**61.98B**	**0.92Aa**	**0.83**	**0.40b**	**1.52**	**0.84Ab**	**0.54**
	SD	—	0	0.12	0.18	0.61	17	0.07	0.11	0.3	1.02	0.12	0.15
	SE	—	0	0.03	0.05	0.16	4.54	0.02	0.03	0.08	0.27	0.03	0.04

**S**_**air-oven**_	**Mean**	—	**0**	**0.00Bb**	**0.85a**	**0.87**	**98.04A**	**0.72B**	**0.73**	**1.13a**	**1.16**	**0.54B**	**0.53**
	SD	—	0	0.07	0.47	0.48	3.85	0.15	0.15	0.91	0.9	0.22	0.22
	SE	—	0	0.02	0.12	0.13	1.03	0.04	0.04	0.24	0.24	0.06	0.06
	F	—	‡‡‡	5.971	7.159	1.007	43.655	12.007	3.065	5.059	2.525	12.168	2.336
	**Sig**	—	—	**0.005** ^**6**^	**0.002** ^**6**^	**0.375**	**0** ^**6**^	**0** ^**6**^	**0.058**	**0.011** ^**5**^	**0.093**	**0** ^**6**^	**0.11**

^1^S_air-and-oven_ = samples including both air-dried and oven-dried *A millefolium*, *S*_oven_ = oven-dried *A millefolium*; *S*_air_ = air-dried *A millefolium*; ^2^sample numbers in the calibration set/sample numbers in the validation set; ^3^a test of variances cannot be performed for c-set bias because the sum of weights is zero; ^4^consistency = (SEE/SEP)*∗*100; ^5^the mean difference is significant at the 0.05 level; ^6^the mean difference is significant at the 0.01 level.

**Table 5:** Correlation analysis between the 14 compounds (Table 1) in *A. millefolium.*

Compound	Parameters	1	2	3	4	5	6	7	8	9	10	11	12	13	14
**1**	*R* ^2^	1	—	—	—	—	—	—	—	—	—	—	—	—	—
	Sig	—	—	—	—	—	—	—	—	—		—	—	—	—
**2**	*R* ^2^	0.70^*∗∗*^	—	—	—	—	—	—	—	—	—	—	—	—	—
	Sig	0	—	—	—	—	—	—	—	—	—	—	—	—	—
**3**	*R* ^2^	0.75^*∗∗*^	0.18	—	—	—	—	—	—	—	—	—	—	—	—
	Sig	0	0.29	—	—	—	—	—	—	—	—	—	—	—	—
**4**	*R* ^2^	−0.41^*∗∗*^	−0.28	−0.27	—	—	—	—	—	—	—	—	—	—	—
	Sig	0.01	0.09	0.1	—	—	—	—	—	—	—	—	—	—	—
**5**	*R* ^2^	0.67^*∗∗*^	0.60^*∗∗*^	0.32^*∗*^	−0.02	—	—	—	—	—	—	—	—	—	—
	Sig	0	0	0.05	0.88	—	—	—	—	—	—	—	—	—	—
**6**	*R* ^2^	0.14	0.52^*∗∗*^	−0.28	−0.18	0.21	—	—	—	—	—	—	—	—	—
	Sig	0.41	0	0.09	0.28	0.21	—	—	—	—	—	—	—	—	—
**7**	*R* ^2^	0.57^*∗∗*^	0.27	0.49^*∗∗*^	−0.38^*∗*^	0.71^*∗∗*^	0.2	—	—	—	—	—	—	—	—
	Sig	0	0.1	0	0.02	0	0.24	—	—	—	—	—	—	—	—
**8**	*R* ^2^	−0.09	0	−0.04	0.92^*∗∗*^	0.25	−0.12	−0.14	—	—	—	—	—	—	—
	Sig	0.59	0.98	0.81	0	0.12	0.48	0.41	—	—	—	—	—	—	—
**9**	*R* ^2^	0.72^*∗∗*^	0.34^*∗*^	0.64^*∗∗*^	−0.19	0.71^*∗∗*^	−0.01	0.74^*∗∗*^	0.1	—	—	—	—	—	—
	Sig	0	0.04	0	0.24	0	0.95	0	0.57	—	—	—	—	—	—
**10**	*R* ^2^	0.53^*∗∗*^	0.58^*∗∗*^	0.1	−0.02	0.75^*∗∗*^	0.37^*∗*^	0.46^*∗∗*^	0.23	0.56^*∗∗*^	—	—	—	—	—
	Sig	0	0	0.55	0.91	0	0.02	0	0.16	0	—	—	—	—	—
**11**	*R* ^2^	0.67^*∗∗*^	0.33^*∗*^	0.51^*∗∗*^	−0.14	0.69^*∗∗*^	−0.09	0.58^*∗∗*^	0.14	0.87^*∗∗*^	0.67^*∗∗*^	—	—	—	—
	Sig	0	0.04	0	0.4	0	0.6	0	0.4	0	0	—	—	—	—
**12**	*R* ^2^	0.88^*∗∗*^	0.59^*∗∗*^	0.62^*∗∗*^	−0.37^*∗*^	0.80^*∗∗*^	0.15	0.76^*∗∗*^	−0.05	0.83^*∗∗*^	0.72^*∗∗*^	0.79^*∗∗*^	—	—	—
	Sig	0	0	0	0.02	0	0.37	0	0.76	0	0	0	—	—	—
**13**	*R* ^2^	0.55^*∗∗*^	0.56^*∗∗*^	0.21	−0.31	0.48^*∗∗*^	0.35^*∗*^	0.37^*∗*^	−0.12	0.53^*∗∗*^	0.45^*∗∗*^	0.54^*∗∗*^	0.53^*∗∗*^	—	—
	Sig	0	0	0.22	0.06	0	0.03	0.02	0.48	0	0.01	0	0	—	—
**14**	*R* ^2^	0.18	0.55^*∗∗*^	−0.34^*∗*^	0.04	0.34^*∗*^	0.39^*∗*^	−0.1	0.17	0.13	0.40^*∗∗*^	0.22	0.15	0.43^*∗∗*^	1.00
	Sig	0.28	0	0.04	0.81	0.04	0.02	0.55	0.32	0.42	0.01	0.19	0.36	0.01	—

^*∗∗*^Correlation significant at the 0.01 level (2-tailed); ^*∗*^correlation significant at the 0.05 level (2-tailed).

**Table 6:** PCA of the 14 compounds and total explained variance.

Factor	Initial eigenvalues			Extraction sum of the loadings		
	Total	% of explained variance	Cumulative % of explained variance	Total	% of explained variance	Cumulative % of explained variance
1	6.54	46.72	46.72	6.54	46.72	46.72
2	2.35	16.76	63.48	2.35	16.76	63.48
3	2.1	14.96	78.44	2.1	14.96	78.44
4	0.85	6.06	84.5	0	0	0
5	0.72	5.17	89.67	0	0	0
6	0.5	3.56	93.23	0	0	0
7	0.35	2.51	95.75	0	0	0
8	0.27	1.92	97.66	0	0	0
9	0.1	0.72	98.38	0	0	0
10	0.08	0.59	98.97	0	0	0
11	0.07	0.47	99.43	0	0	0
12	0.05	0.33	99.76	0	0	0
13	0.02	0.18	99.94	0	0	0
14	0.01	0.06	100.00	0	0	0

## Data Availability

The data used to support this study are available from the corresponding author upon request.
